# Differential metabolic responses in bold and shy sea anemones during a simulated heatwave

**DOI:** 10.1242/jeb.244662

**Published:** 2024-02-07

**Authors:** Daniel K. Maskrey, Shaun S. Killen, Lynne U. Sneddon, Kathryn E. Arnold, David C. C. Wolfenden, Jack S. Thomson

**Affiliations:** ^1^ Department of Earth, Ocean and Ecological Sciences, School of Environmental Sciences, Nicholson Building, University of Liverpool, Liverpool L69 3GP, UK; ^2^ Institute of Biodiversity, Animal Health & Comparative Medicine, Graham Kerr Building, University of Glasgow, Glasgow G12 8QQ, UK; ^3^ Department of Biological & Environmental Sciences, University of Gothenburg, Box 463, SE-405 30 Gothenburg, Sweden; ^4^ Department of Environment and Geography, Wentworth Way, University of York, Heslington, York YO10 5NG, UK

**Keywords:** Climate change, Boldness, Metabolism, Pace of life, Marine invertebrate

## Abstract

As climate change-induced heatwaves become more common, phenotypic plasticity at multiple levels is a key mitigation strategy by which organisms can optimise selective outcomes. In ectotherms, changes to both metabolism and behaviour can help alleviate thermal stress. Nonetheless, no study in any ectotherm has yet empirically investigated how changing temperatures affect among-individual differences in the associations between these traits. Using the beadlet anemone (*Actinia equina*), an intertidal species from a thermally heterogeneous environment, we investigated how individual metabolic rates, linked to morphotypic differences in *A. equina*, and boldness were related across changing temperatures. A crossed-over design and a temporal control were used to test the same individuals at a non-stressful temperature, 13°C, and under a simulated heatwave at 21°C. At each temperature, short-term repeated measurements of routine metabolic rate (RMR) and a single measurement of a repeatable boldness-related behaviour, immersion response time (IRT), were made. Individual differences, but not morphotypic differences, were highly predictive of metabolic plasticity, and the plasticity of RMR was associated with IRT. At 13°C, shy animals had the highest metabolic rates, while at 21°C, this relationship was reversed. Individuals that were bold at 13°C also exhibited the highest metabolic rates at 21°C. Additional metabolic challenges during heatwaves could be detrimental to fitness in bold individuals. Equally, lower metabolic rates at non-stressful temperatures could be necessary for optimal survival as heatwaves become more common. These results provide novel insight into the relationship between metabolic and behavioural plasticity, and its adaptive implications in a changing climate.

## Teaser

**Summary:** Plastic changes in metabolic rates are associated with boldness in beadlet anemones, such that different personalities show opposite metabolic patterns under a simulated heatwave, as compared with a non-stressful temperature.

## INTRODUCTION

The pace-of-life syndrome hypothesis (POLS; Réale et al., 2010) posits that organisms must trade-off long-term survival against short-term reproductive success, leading to predictable correlations between risk-related or metabolically costly behaviour and energetic physiology. POLS predicts that individuals with more risk-prone, ‘bolder’ personalities (consistent behavioural differences among individuals; Sih et al., 2004), should show ‘faster’ physiological characteristics, such as a high metabolic rate, that are conducive to higher early fecundity and growth, but not to long-term survival (Montiglio et al., 2018; Réale et al., 2010). It is well established that life-history trade-offs can drive the maintenance of personality types (Wolf and McNamara, 2012; Wolf and Weissing, 2010); bold individuals often place themselves at greater risk of mortality than shy conspecifics, but benefit from riskier lifestyles via increased foraging opportunities and more energetic scope for reproduction (Smith and Blumstein, 2008). POLS simply extends these trade-offs by predicting that the physiology of bolder individuals should be primed to maintain high levels of risky activity, exploration and foraging, even under periods of heightened environmental stress (Montiglio et al., 2018; Réale et al., 2010). As such, according to POLS, bolder animals should exhibit reduced physiological stress reactivity, faster growth and higher metabolic rates than shyer conspecifics (Biro and Stamps, 2010). The relationships proposed by POLS could be of great value to conservationists, indicating the life-history strategies present in a given population and informing whether some behavioural phenotypes might be more susceptible to novel selective pressures than others.

Evidence for POLS is mixed, with studies finding positive (Polverino et al., 2018), negative (Le Galliard et al., 2013) and inconclusive associations (Killen et al., 2012) between metabolic rate and boldness. As such, a recent meta-analysis concluded that empirical evidence for POLS is weak (Royauté et al., 2018). However, no studies have yet investigated how individual variation in the scope and nature of phenotypic plasticity to environmental fluctuation might affect relationships between bioenergetics and boldness (Killen et al., 2013; Montiglio et al., 2018). ‘Activational’ phenotypic plasticity (Snell-Rood, 2013), an animal's ability to rapidly alter aspects of its phenotype in response to acute environmental change (Seebacher et al., 2015; Stamps, 2016), could be crucial to understanding these relationships under a rapidly changing climate, where extreme weather events are becoming increasingly common (IPCC, 2013, 2018). Activational plasticity can have selective advantages, helping many species remain robust to fluctuating environments (Snell-Rood, 2013), and disadvantages, as maintaining scope for short-term plasticity is energetically costly (Dall et al., 2004). This leads to variation within populations in the ability of different individuals to deal with acute environmental changes (Dingemanse and Wolf, 2013) and could thus influence relationships between metabolism and boldness, and their selective implications under different environmental scenarios.

Although activational plasticity does not itself form part of POLS (Montiglio et al., 2018), the relationship between metabolism and behaviour is likely to be both species and context dependent (Killen et al., 2012). In mosquitofish, for example, lineages from more stochastic environments grow faster, but also exhibit lower metabolic rates and are shyer than those from stable environments (Polverino et al., 2018). Among-individual variation in plasticity may be an especially important driver of relationships between behaviour and metabolism in heterogeneous environments, where the coexistence of different plastic strategies can be driven by spatial and temporal variation both in normal environmental conditions and in how much those conditions fluctuate (Dingemanse and Wolf, 2013; Wolf and McNamara, 2012). Despite the clear need to incorporate activational plasticity into investigations of POLS and its selective implications, we currently do not have any empirical information on how short-term environmental changes, and the overall variability of an animal's environment, might affect the relationships between metabolism and behaviour.

When investigating how acute environmental changes affect these relationships, temperature is a particularly important environmental variable to consider. As climate change leads to higher average temperatures and increased frequency of heatwaves (IPCC, 2013, 2018), animal populations are being placed under substantial, novel selective pressures (Parmesan, 2006). Thermal physiology and thermal preferences are especially important drivers of associations between metabolism and behaviour in ectotherms (Abram et al., 2017), where many physiological processes, including metabolic rate, are intrinsically linked to environmental temperatures (Seebacher et al., 2015). In ants, for example, colonies from warmer environments are more likely to show the positive associations among energetically costly or risk-related behaviours that are predicted by POLS (Segev et al., 2017). Adult parasitic wasps with a preference for low temperatures are another example, exhibiting high metabolic rates, low foraging efficiency and short lifespans when exposed to high temperatures (Le Lann et al., 2011). As an acute thermal stressor to which they are unable to immediately physiologically adjust, climate change-induced heatwaves present ectotherms with a different set of ecological challenges compared with chronic temperature change (Vajedsamiei et al., 2021), and many species rely heavily on behavioural plasticity to address these challenges (Abram et al., 2017). Nonetheless, the effect of acute temperature rises on the relationship between ectothermic physiology and behaviour has yet to be investigated.

The beadlet anemone, *Actinia equina*, lives across a gradient of shore heights in the spatially and temporally heterogeneous intertidal zone (Allcock et al., 1998). Although *A. equina* is particularly robust to temperature stress (Griffiths, 1977), making it an ideal organism with which to investigate the effects of thermal perturbation without risk of mortality, the heterogeneous nature of its environment leads it to show ranges of thermal preferences within single populations (Navarro et al., 1981). Anemones living further from the low-tide line (higher up the shore), experience increased exposure to stressful temperatures (Brahim et al., 2019) and greater thermal variation than those living lower down the shore (Chapperon et al., 2016). Multiple genetically distinct morphotypes have developed which favour different shore heights; the red morphotype is associated with the high shore, and is thus predicted to deal more effectively with temperature extremes than the green morphotype, which is associated with the low shore (Allcock et al., 1998). Morphotypes further display consistent differences in boldness-related behaviours. Of particular relevance could be immersion response time (IRT), which not only displays among-individual variation within and across environments but also may be closely associated with an anemone's metabolic rate (Maskrey et al., 2020, 2021). IRT is a risk-related behaviour, which measures how long after a simulated tide cycle it takes an anemone to re-extend its feeding tentacles and resume foraging. Because *A. equina* is unable to feed when the tide is out, more protracted tentacle retraction upon immersion will lead to an animal having less time to forage and thus less energy for metabolic processes. Further, tentacle extension in *A. equina* is inherently linked to respiratory efficiency and gas exchange (although anemones do continue to respire with their tentacles retracted or when exposed; Griffiths, 1977; Navarro et al., 1981), so spending longer with tentacles retracted can be highly costly in the face of metabolic challenges (Griffiths, 1977). Conversely, while its primary function is to support essential metabolic processes and gas exchange, tentacle extension is also risky, placing anemones under increased threat of predation (Edmunds et al., 1974, 1976). Given that anemones are still able to respire with their tentacles retracted (Griffiths, 1977; Navarro et al., 1981), and thus tentacle extension is not perfectly correlated with routine metabolic rate (RMR, the metabolic rate of an animal undergoing normal activity), tentacle extension behaviour should represent a trade-off. If an individual is more cautious to predation risk, it is likely to face the costs of reduced efficiency of gas exchange and reduced foraging opportunities but may survive longer in the face of predators. As such, general tentacle extension behaviour, and in turn IRT, should act as a useful gauge of boldness. This trade-off should be particularly pronounced at high temperatures, which drive increases in metabolic demand (Abram et al., 2017) but are also likely to place anemones under greater risk of predation from other ectotherms (e.g. Twardochleb et al., 2020; Yamane and Gilman, 2009). Relationships between IRT and metabolic rate at different temperatures should thus provide clear indications of whether *A. equina* follows POLS and enable prediction of the metabolic responses of different individuals to thermal extremes.

In this study, we investigated for the first time how relationships between behavioural phenotypes and metabolic rates were influenced by different temperatures, both within and among individuals. We measured the associations between RMR (Metcalfe et al., 2016; Velasque and Briffa, 2016) of two morphotypes of *A. equina* and their IRTs at two temperatures using a crossed-over temperature design, where one temperature was non-stressful and the other simulated a heatwave. We also incorporated a temporal control where the temperature remained non-stressful throughout. Importantly, all experimental individuals had their IRTs and RMRs measured at both temperatures, allowing us to use Bayesian multivariate methodologies to investigate associations between IRT and RMR both within and across environments. We expected that RMR would show a general increase across all individuals at high temperatures and that bolder animals, exhibiting shorter IRTs, would have higher RMRs at both temperatures, as predicted by POLS. As both metabolic demand and predation risk should be exacerbated at high temperatures, which should theoretically intensify any trade-off between the efficiency of gas exchange and foraging, and risk taking, we further predicted that this relationship would be stronger at high temperatures. We also predicted that the degree of change in RMR between temperatures would vary at individual and morphotypic levels, with discrepancies being influenced by the red morphotype's tolerance for higher, more variable temperatures than the green.

## MATERIALS AND METHODS

### Experimental schedule

Data collection was carried out between January and March 2020 over the course of four, 9 day data collection blocks. Each block began with 3 days of anemone, *Actinia equina* (Linnaeus), collection from rocky sea defences at the top of the beach at New Brighton, UK (latitude: 53.4400, longitude: −3.0565). Five animals were collected on each day, split between the red and green morphotypes, such that three of one morphotype and two of the other were collected. This uneven split, necessary because of 15 being the maximum number of individuals that could be investigated per block in our respirometry apparatus, was randomised and evened out over the course of the study. Overall, 30 anemones of each morphotype were used. Collection and identification of morphotypes were carried out using previously described methods (Maskrey et al., 2020). Anemones were size matched at the point of collection as far as was possible by measuring pedal disc diameter (PDD). Because the raw RMR (Metcalfe et al., 2016; Velasque and Briffa, 2016) of larger individuals of all species is higher than that of smaller individuals (Glazier, 2005), and thus easier to accurately detect, only anemones with a PDD of over 20 mm, the largest individuals in the population, were selected. Further, because pilot work found the measurement of wet mass to be highly invasive and cause significant damage to anemones, and because the volume of *A. equina* is not static (Griffiths, 1977) so cannot be reliably measured, this size matching was also used to minimise any effect of anemone volume on metabolic results.

Each group of five anemones was returned to the laboratory, again using previously described methods (Maskrey et al., 2020), and left to acclimate to their surroundings for 48 h. Anemones were housed in a 13±1°C temperature-controlled room on a 12 h:12 h day:night cycle. Each anemone was placed in a 7×15 cm plastic cup containing drainage holes and a rock to which the anemone could adhere. Cups were situated in one of two 80×45×40 cm tanks filled to a depth of ∼12 cm with artificial seawater, at a salinity of 34±1 ppt (RO water with Tropic Marin Pro Reef Salt, Germany). One of these tanks, which housed 10 individuals, was maintained at the ambient temperature of 13°C (±1°C), while the other, which housed 5 individuals, was maintained at 21°C (±1°C): 13°C was chosen as a suitable non-stressful temperature because it is well within the normal range of ambient temperatures *A. equina* would experience on the shore at New Brighton (Maskrey et al., 2021); 21°C, meanwhile, falls near the thermal maximum for this species and is well above the normal temperature range experienced in this region (Maskrey et al., 2020). As such, exposure to three consecutive days at this temperature was deemed an appropriate method by which to simulate the effects of an atmospheric heatwave on the intertidal zone (Perkins and Alexander, 2013). Tanks were situated in two flow-through systems, which each contained three tanks. Every tank contained anemones at least once and the order in which tanks were used was randomised across blocks. Across the four blocks, each system was maintained at each temperature twice, and the order of this was randomised. Tanks were retained at a single temperature within blocks. Water quality (salinity, pH, nitrate, nitrite, ammonia) and water temperature in holding tanks were regularly recorded and full water changes in each system were carried out before each block.

After 24 h of the 48 h acclimation period, anemones were fed *ad libitum* on defrosted *Artemia* (Monkfield Nutrition, Ely, UK). This gave anemones time to bind to the substrate and limited variation among individuals in levels of satiation, whilst also allowing a period of 24 h without food before metabolic experimentation commenced. After the full 48 h, each group of five anemones was first subject to an IRT measurement at ∼17:00 h, using the anemones' natural response to changes in tides to measure boldness. IRTs have previously been shown to be repeatable within contexts (*r*=0.26–0.4), and show significant among-individual variation in how they change across temperatures (Maskrey et al., 2020, 2021). Similarly, risk-related tentacle extension behaviour has been shown to exhibit both repeatability and among-individual variation in plasticity in *A. equina* and other anemone species (Briffa and Greenaway, 2011; Hensley et al., 2012; Rudin and Briffa, 2012). As such, although not taking repeated measurements of a trait can give rise to statistical concerns in some cases (Brommer, 2013), a single measurement of IRT was taken before the commencement of metabolic testing, both because of the weight of previous evidence indicating that variation in IRT is associated with among-individual differences and because of logistical constraints. Cups containing anemones were removed from holding tanks and drained. Anemones were left emersed for half an hour, before being re-immersed in their holding tank. After re-immersion, IRT, defined as the length of time to re-extend feeding tentacles fully (Maskrey et al., 2020), was recorded by two GoPro Hero 4 (GoPro Inc., San Mateo, CA, USA) cameras mounted directly above the tank, taking time-lapse photographs every 30 s; 50 min of footage was recorded, of which the 45 min immediately after each individual's immersion was used to determine IRT. The number of photos an anemone took to re-extend its tentacles was recorded by D.K.M. (blinded to treatment) and converted into seconds. Anemones which showed no complete response within 45 min were given a maximum value of 2700 s (Maskrey et al., 2020).

After their evening IRT measurement, anemones were gently separated from their pebbles and transferred to one of six freshly sterilised (with bleach-water and rinsed with fresh water) 14×6 cm, 425 ml glass intermittent-flow respirometry chambers sealed with polypropylene lids (IKEA, Älmhult, Sweden) and each containing a 2.5 g magnetic stir bar with which anemones could not interact. One of these chambers was designated as the blank, and thus remained empty. For identification purposes, anemones of the same morphotype were grouped in adjacent chambers, but where the morphotypes were, and which chamber was designated as the blank, was randomised. Chambers were situated in a temperature-controlled respirometry tank (67×46×38 cm) filled to a depth of 20 cm with freshly made artificial seawater (salinity 34±1 ppt). The seawater was heated or chilled to the appropriate temperature (±0.3°C) prior to anemone introduction. Chambers were also subject to blank measurements prior to the introduction of anemones to ensure that only negligible background respiration was present; where background was detected, chambers were re-sterilised to ensure minimal microbial activity. After their introduction, anemones were left for 12 h overnight to acclimate and attach to their chamber. We used automated intermittent-flow respirometry (Svendsen et al., 2016) to measure aquatic RMR, so this 12 h period also served to acclimate anemones to the intermittent-flow cycle. Measurements could not be conducted throughout the night because of difficulties with having the magnetic stir bars move continuously for 12 h. The intermittent-flow cycle consisted of a 3 min water flush period and a 38 min closed period where oxygen consumption was measured. This measurement period was shorter than those used in previous *A. equina* respirometry studies (e.g. Navarro et al., 1981), but with this volume of chamber, our pilot investigations found it produced sufficient data with which to measure oxygen consumption, while allowing more repeated metabolic measurements to be taken each day. After this 12 h overnight acclimation period, experimentation was carried out for 4 h in the morning and 4 h in the afternoon, with a brief (∼10 min) break period between the two where any small bubbles that had formed were removed from chambers. During trials, aquatic oxygen concentration within chambers never fell below 80% saturation. Four metabolic slopes of oxygen consumption were produced for each anemone at each time of day (for full descriptions and checklist, see Supplementary Materials and Methods, ‘1.1 Metabolic apparatus and additional measurement detail’ and Table S1; Killen et al., 2021).

At the end of their first day of testing, groups were transferred back to one of the two holding tanks and the whole cycle was repeated, beginning with another 48 h acclimation period. To account for temporal variation and treatment order effects, we utilised a crossed-over design (Briffa et al., 2013) and a temporal control (White and Briffa, 2017). Within blocks, each group of five anemones was designated to one of three treatment groups, which were subjected to repeated RMR measurements and a single IRT measurement at each temperature. Each treatment had an overall sample size of 10 red and 10 green individuals: (1) The temporal control (L–L) group was housed and tested at 13°C throughout; (2) the low temperature to high temperature group (L–H) was first housed and tested at 13°C, then at 21°C; (3) the high temperature to low temperature group (H–L) was first housed and tested at 21°C, then at 13°C.

**Fig. 1.**
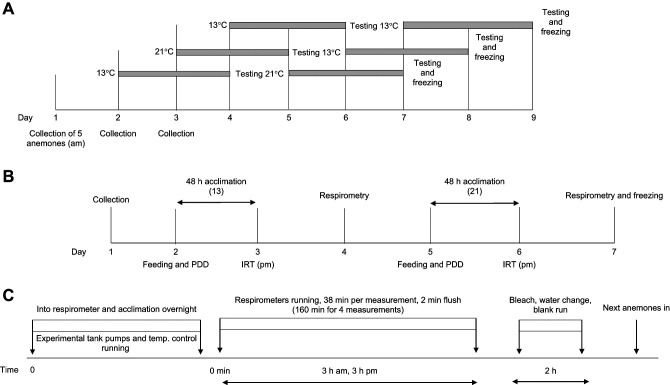
**Schematic diagram of the experimental design.** (A) A full example block schedule (where grey bars indicate acclimation periods), (B) the schedule for a low to high temperature (L–H) group (kept initially at 13°C and changed to 21°C on day 4) and (C) an example respirometry schedule. IRT, immersion response time; PDD, pedal disc diameter.

After the second round of metabolic testing, anemones were placed directly into a −20°C freezer in individually labelled plastic bags, to be stored for later drying and weighing. This method of euthanasia added an extra step compared with previous studies of *A. equina*, where anemones were dried from live (e.g. Navarro et al., 1981; Rudin and Briffa, 2012). Fig. 1 provides a visualisation of this schedule.

### Morphological measurement

To measure the dry mass of each individual, frozen anemones were placed in a Carbolite (Hope Valley, UK) CWF100 muffle furnace, maintained at 110°C for 90 h. Dry anemones were then weighed using a Sartorius (Stonehouse, UK) R2000 balance scale, accurate to the nearest hundredth of a milligram.

### Statistical analysis

The question of how behavioural and other phenotypic traits covary with one another within and across environments is multivariate by its nature. Multiple traits can be influenced by the same set of fixed effects, including environmental variation, while simultaneously covarying with one another. While univariate modelling approaches facilitate investigating single traits (Nakagawa and Schielzeth, 2010), they are not appropriate for investigating the associations between different traits (Houslay and Wilson, 2017), giving rise to anti-conservative correlation estimates and spurious conclusions. Simple correlational analyses using raw, rather than modelled, data are also of limited value when investigating relationships between phenotypic traits as they do not account for the complexity of the relationships in question (Nakagawa and Schielzeth, 2010). Many researchers in behavioural ecology and quantitative genetics have thus turned to multivariate mixed effects modelling to better describe these multivariate questions (Cleasby et al., 2015; Houslay and Wilson, 2017; Mitchell et al., 2016). While it is possible to fit multivariate models using a frequentist framework, methods utilising free software or statistical packages lack flexibility (e.g. lme4: Bates et al., 2015), and relatively more flexible frequentist packages require paying a subscription (e.g. ASReml-r v.4.2: https://asreml.kb.vsni.co.uk/knowledge-base/asreml_r_documentation/). By contrast, multiple free, flexible Bayesian packages and software exist for fitting mixed effects models across multiple response variables or across multiple levels of variance (e.g. MCMCglmm: https://CRAN.R-project.org/package=MCMCglmm; JAGS: https://mcmc-jags.sourceforge.io/; Stan: Carpenter et al., 2017). By fitting Bayesian models over uninformative priors as we do in this study, study data drive modelled posterior distributions, meaning that the information and estimates contained in those distributions are functionally the same as those contained in frequentist models (Cleasby et al., 2015; Houslay and Wilson, 2017). Moreover, Bayesian methodologies are more robust to small sample sizes, or violations of the assumptions of frequentist modelling approaches (McNeish, 2016). Bayesian modelling has become standard practice when investigating associations between multiple phenotypic traits within or across environments (e.g. Briffa et al., 2013; Jolles et al., 2019; Maskrey et al., 2021; Mitchell et al., 2020), and current advancements in these approaches use predominantly Bayesian frameworks (Mitchell et al., 2016; Mitchell and Houslay, 2021; Prentice et al., 2020). We utilised these methods, exploring associations between traits at multiple phenotypic levels (i.e. behavioural, as IRT, and physiological, as RMR), here.

#### Calculation of RMR

Metabolic data were analysed using the respR package (Harianto et al., 2019) in R version 3.6.2 or later (http://www.R-project.org/). A minimum of two slopes were extracted for every chamber for both morning and afternoon sets of readings, providing up to six measurements of oxygen consumption per chamber, per day. The first 41 min cycles in both the morning and afternoon were always excluded, as preliminary data indicated that RMR settled during the first cycle, such that values were different from subsequent slopes in a sampling repeat. After a wait period before recording of at least 1 min to ensure only the linear portion of slopes were measured (Svendsen et al., 2016), the *r*^2^ of recorded oxygen consumption slopes for all anemones fell above a threshold of 0.9. For each repeated measure, slopes from blank chambers were used to calculate background-adjusted whole-organism oxygen consumption (RMR) by subtracting blank oxygen consumption values from the values of each experimental chamber. Raw blank-adjusted measurements were converted to provide final whole-organism short-term RMR estimates in milligrams of oxygen per hour (mg O_2_ h^−1^; Supplementary Materials and Methods, ‘1.2 Example slopes and conversion’). Each of these slopes was treated as a repeated measure in subsequent analyses.

#### Bayesian analyses

All individual-level models were run within a Bayesian Markov chain Monte Carlo framework using the R package MCMCglmm (https://CRAN.R-project.org/package=MCMCglmm). See Supplementary Materials and Methods, ‘1.3 Statistical detail’, for details of general model specification and convergence checks, additional analytical details, and discussion of a confirmatory analysis into the relationship between RMR and dry mass.

#### Repeatability and plasticity of RMR

Before investigating relationships between RMR and IRT across temperatures, we first sought to confirm the presence of among-individual variation in RMR and in its plasticity, a pattern previous research has already shown in IRT (Maskrey et al., 2020, 2021). We also sought to investigate the influence of morphotypic differences on RMR. To achieve this, a random slopes model was run (see Supplementary Materials and Methods, ‘1.3 Statistical detail’, for details of comparison with the equivalent random intercepts model). RMR (*z*-transformed) was the response variable and morphotype, temperature, treatment, dry mass (*z*-transformed), sampling occasion, sampling day (i.e. whether it was an individual's first or second day of metabolic testing), and data collection block (*z*-transformed) were all included as fixed effects. Because data collection block was temporally directional, and to improve model fits and simplify outputs, it was included in this and subsequent models as a continuous variable. This was denoted by the number of days between the start of the first block and the start of the block in question (0, 12, 28 and 41, respectively). *z*-Transformation of continuous variables, which centres data on zero based on how many standard deviations away from the mean each data point falls, was carried out in this and subsequent models to improve model convergence by placing continuous variables on the same scale (Houslay and Wilson, 2017). Temperature was allowed to interact, separately, with morphotype and treatment order. Previous iterations of univariate random regressions showed no significant interaction between temperature and dry mass in relation to RMR (*P*=0.111). This interaction was thus removed from the models as it was not of direct interest, unlike those between temperature and morphotype or temperature and treatment order. A random slope effect, accounting for individual variation and the interaction between individual identity and metabolic plasticity to temperature (individual×environment, I×E), was specified as the random effect. The inclusion of temporal control data in the model, alongside including sampling occasion as a fixed effect, allowed robust control for both treatment order- and time-related effects.

To investigate whether individual differences in metabolic rate were consistent across the two temperatures, adjusted cross-context repeatability was calculated. To test the explanatory importance of individual variation in determining metabolic plasticity to temperature, the temperature-driven I×E, or random slope effect estimate, was extracted. Finally, to explore how an individual's metabolic rate at 13°C related to their metabolic plasticity to temperature, the correlation between individual intercepts at 13°C and their random slopes between 13°C and 21°C was calculated. Statistical significance was inferred for fixed effects and covariance terms where 95% credible intervals did not cross zero. For repeatability and random slope effect estimates, significance was inferred where 95% credible intervals were not close to zero and histograms of the term's posterior estimates were not pushed up against zero (Maskrey et al., 2020).

#### The relationship between RMR and IRT

We explored several approaches to investigate the relationship between RMR and IRT, how it might relate to POLS at different temperatures, and whether boldness at 13°C or 21°C could be used to predict RMR at 21°C, whilst accounting for censored IRT data. Initially, we used a bivariate analysis with the brms package in Stan, using a Gaussian distribution for rate measures and a Weibull distribution for the censored IRT data; however, the software was unable to fit the appropriate model. We were also unable to develop a model in MCMCglmm that allowed for both a multi-variable response and censored observations. As such, we used a Gaussian distribution for IRT without censoring; whilst there are problems associated with this approach, described below, current approaches struggle to deal with these data (as explored in Stamps et al., 2012), and the approach used had been previously justified (see Maskrey et al., 2021).

Two separate datasets were used. The ‘13°C dataset’ included all values measured at 13°C, including 13°C values from both the control and crossed-over temperature treatments. The ‘crossover dataset’ included all values measured in the crossed-over temperature treatments at either temperature. A bivariate mixed effects model was run on the 13°C dataset to determine the covariance between the two traits at 13°C, with RMR and IRT (both *z*-transformed) set as response variables. For the crossover dataset, a multivariate model was run where the four response variables were *z*-transformed RMR and IRT at each temperature. For both models, morphotype, dry mass (*z*-transformed), treatment, sampling occasion and data collection block were all fixed effects, with individual identity included as a random effect. Residual (within-individual) covariance was not identifiable, both because IRT and RMR were not measured at the same time in any treatment, and because only one IRT measurement per individual was modelled within each temperature for crossed-over treatments. As such, values within the residual variance–covariance matrices of each model were therefore fixed at 1. After the appropriate model checks had been carried out, among-individual correlations between IRT and RMR within each temperature and between temperatures, alongside associated 95% credible intervals (CIs), were extracted. Statistical significance was inferred where 95% CIs did not cross zero. This analysis was split into a bivariate and a multivariate component to allow the incorporation of the control data into the 13°C correlation estimate. Including control data in the bivariate model was especially important to account for temporal effects, as 13°C experimental data only covered either the first or second sampling day, depending on treatment. Conversely, while control data were not included in the multivariate model, the nature of the crossover dataset, incorporating both sampling days for both treatments, means that the multivariate model should still have been robust to both treatment order- and time-related effects.

### Ethical note

No licences or ethical approval was required to carry out this study as this species is not currently protected under UK legislation. Nonetheless, anemones were treated humanely, temperatures to which anemones were exposed fell within their tolerance ranges (Maskrey et al., 2020), and no mortality occurred during the experimental periods.

## RESULTS

### Repeatability and plasticity of RMR

**Fig. 2.**
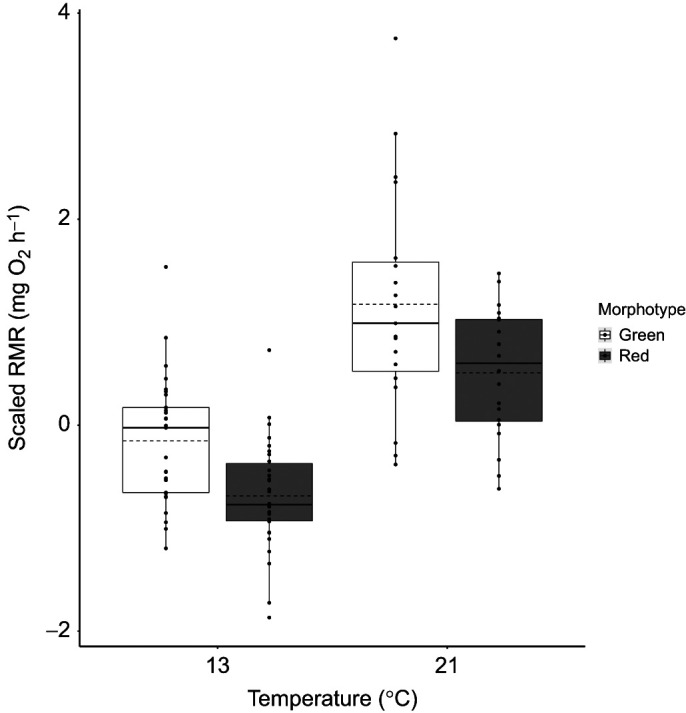
**Variation in predicted mean routine metabolic rate (RMR) between red and green morphotypes of the beadlet anemone (*Actinia equina*) at 13°C and 21°C.** Data are scaled to standard deviation units and centred on the mean at zero for all individuals (*n*=60), derived from a Bayesian random slopes analysis. Boxes denote the median value, with the first and third quartiles forming the box limits; dashed lines denote the mean; whiskers extend to encompass all data or 1.5 times the interquartile range. Any point falling outside the whiskers can be deemed to be an outlier.

There was a general trend across almost all animals for increased RMR at 21°C such that sample mean RMR increased by over 50% from 0.12±0.04 mg O_2_ h^−1^ (mean±s.d.) at 13°C to 0.19±0.06 mg O_2_ h^−1^ at 21°C. This translated into a significant modelled temperature effect on RMR, but the size of this effect was estimated with a large degree of uncertainty (estimate=0.98, 95% CI=0.05, 1.95). Both morphotype and dry mass were also significant predictors of RMR, and neither of these relationships was affected by temperature. The green morphotype exhibited significantly higher mean RMR than the red morphotype at both temperatures (25% higher at 13°C and 21% higher at 21°C; estimate=−0.34, 95% CI=−0.59, −0.10), although Fig. 2 does show a wider range of RMR values for both morphotypes at 21°C. Heavier individuals exhibited significantly higher RMRs than lighter individuals (estimate=0.41, 95% CI=0.29, 0.53). The relationship between data collection block and RMR was also on the verge of significance (estimate=−0.12, 95% CI=−0.24, −0.01), such that animals in later blocks had lower RMRs than those in earlier blocks.

**Fig. 3.**
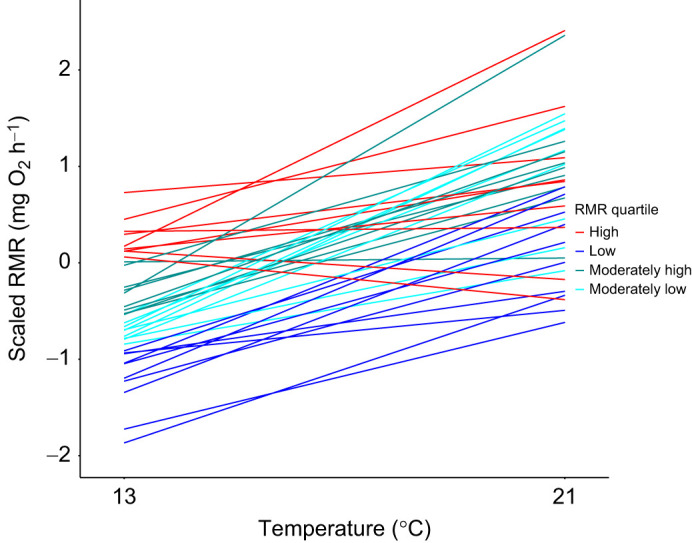
**The change in individual-level posterior mean predictions for RMR between 13°C and 21°C for experimental individuals.** Data are scaled to standard deviation units and centred on the mean at zero. Each line corresponds to a single individual's (*n*=40) predicted RMR at each temperature, coloured based on their RMR quartiles, compared with other experimental individuals, at 13°C. Predictions are derived from a random regression model across 13°C and 21°C run using the full dataset incorporating control individuals (*n*=60). For visualisation purposes, one individual that exhibited especially high scaled estimates at both temperatures (13°C: 1.54 mg O_2_ h^−1^, 21°C: 3.76 mg O_2_ h^−1^), was excluded from the plot.

At the individual level, repeatability estimates indicated that RMR varied consistently among individuals and that some of this variation was retained across temperatures, such that the rank order of individual RMRs at 21°C remained partially similar to the rank order at 13°C. As such, adjusted cross-context repeatability was low, but still significant (*R*_adj_=0.18, 95% CI=0.11, 0.25). This result should be taken with some caution, given that while individuals did show variation in the magnitude of their metabolic slopes within temperatures, within-temperature measurements may have been subject to some level of temporal autocorrelation (Mitchell et al., 2020), leading to a lower cross-context repeatability estimate. Nonetheless, in these data, much of the among-individual variation in repeated RMR measurements was explained by I×E, translating into a highly significant, strong random slope effect (estimate=0.70, 95% CI=0.60, 0.80). Fig. 3 shows that individuals differed greatly in the degree to which their short-term RMR increased as the temperature was raised: 2/40 exhibited lower RMRs at 21°C than at 13°C and 2/40 showed minimal change between the two temperatures. How the RMR of different individuals changed between temperatures was associated with their RMR at 13°C, with individuals that exhibited lower RMRs at 13°C showing larger increases at 21°C than those that exhibited higher RMRs at 13°C. This translated into a significant negative correlation between individual RMRs at 13°C and the gradient of individual random slopes between the two temperatures (*r*=−0.53, 95% CI=−0.70, −0.21).

### The relationship between RMR and IRT

**Fig. 4.**
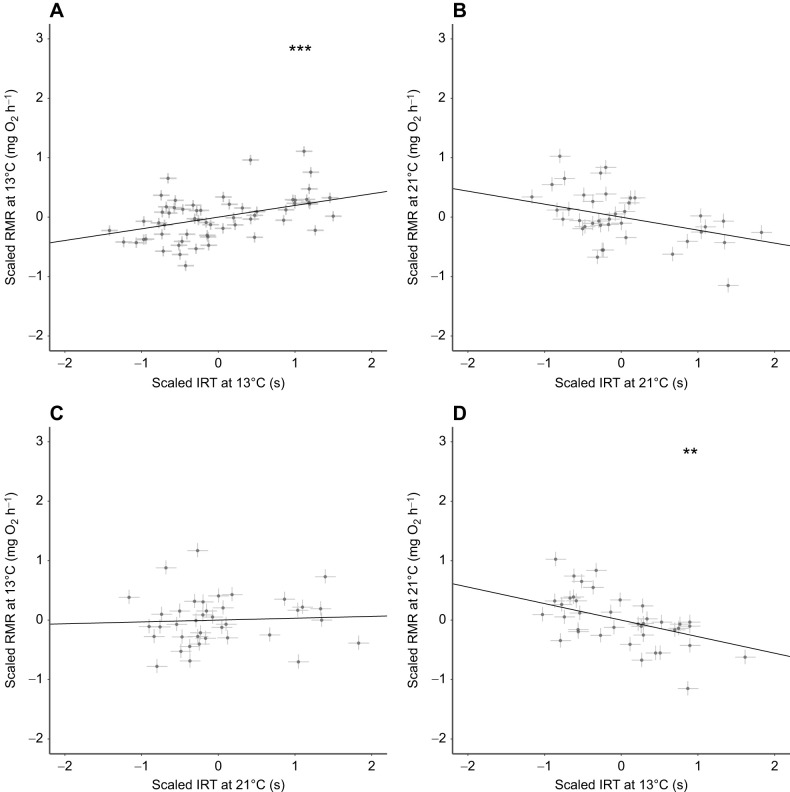
**The relationship between individual-level posterior mode estimates (Bayesian BLUPs) for RMR and immersion response time (IRT) at 13°C and 21°C.** Data are scaled to their respective standard deviation units and centred on their respective means at zero, at (A) 13°C for both traits, (B) 21°C for both traits, (C) 13°C for RMR and 21°C for IRT and (D) 21°C for RMR and 13°C for IRT. ***Statistically significant relationship between the two traits. **Relationship falling at the bound of statistical significance. Estimates are derived from bivariate (A) and multivariate (B–D) Bayesian mixed effects models. Error bars denote 95% credible intervals around each of the traits at each temperature.

RMR was correlated with IRT, and the nature of this correlation was related to environmental temperature such that it did not always follow the assumptions of POLS. At 13°C, shyer individuals (exhibiting longer IRTs) showed higher RMRs than bolder individuals, translating into a moderate, significant positive correlation between IRT and RMR at that temperature (Table 1, Fig. 4A). At 21°C, the pattern of this correlation swapped, such that bolder individuals (with shorter IRTs) at either temperature exhibited higher RMRs at 21°C than individuals that were shyer at either temperature (Figs 4B,D). This correlation, while still of interest, was not significant between IRT and RMR both measured at 21°C (Table 1, Fig. 4B). It was, however, at the bound of significance between IRT at 13°C and RMR at 21°C, as repeated runs of the model found the lower bound of the 95% CI to hover within 0.01 units either side of 0 (Table 1, which shows the results of one such run; Fig. 4D). This indicates that an individual's IRT at 13°C was moderately predictive of its RMR at 21°C. It is worth noting that this significance may have been clearer but for the smaller effective sample size of the crossover dataset (*n*=40). There was no relationship between RMR at 13°C and IRT at 21°C (Table 1, Fig. 4C).

**Table 1.**
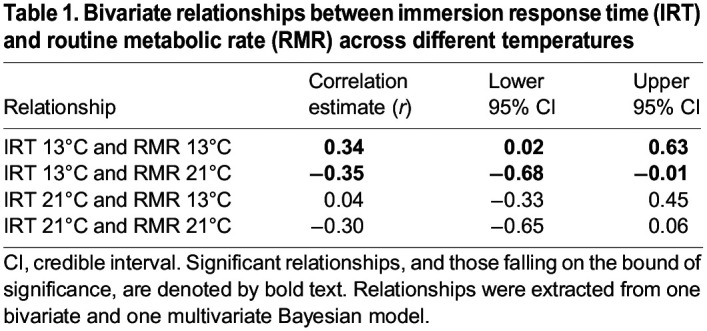
Bivariate relationships between immersion response time (IRT) and routine metabolic rate (RMR) across different temperatures

## DISCUSSION

As the climate warms and marine invertebrates experience extreme temperatures with increasing regularity (IPCC, 2013), the survival of individuals under the greatest metabolic demand at those temperatures may be placed in jeopardy (Montiglio et al., 2018). The POLS hypothesis (Réale et al., 2010), which predicts positive correlations between boldness and metabolic rate, could be used to help indicate those individuals most vulnerable to warming, but how these correlations change when individuals are exposed to ecologically relevant heat stress has not been tested. In this study, we found evidence in beadlet sea anemones for a complex association between RMR and IRT across temperatures, such that the correlations between the two did not follow the assumptions of POLS at a non-stressful temperature of 13°C but did during a simulated heatwave at 21°C. We further showed that individual differences explained most of the variance in how RMR changed between temperatures. If individuals of certain personality types are behaviourally or physiologically more sensitive to temperature stress, using more energy or experiencing reduced foraging opportunities or increased predation risk, then more regular heatwaves could put those individuals at a selective disadvantage.

At a non-stressful temperature, shyer individuals exhibited higher RMRs, a result entirely at odds with POLS. One intuitive explanation for this could be that, at lower temperatures, shyer individuals were investing more than bolder individuals in metabolically costly maintenance processes such as growth, as suggested by the allocation model of resource management (Biro et al., 2020; Careau et al., 2019). Although population-wide studies tend to find faster growth at warmer temperatures in marine invertebrates (reviewed in Angilletta et al., 2004), this rule is not universal (Angilletta and Dunham, 2003; Biro et al., 2020; Careau et al., 2019). In *A. equina*, even very regular feeding regimens are not enough to counteract a loss of body mass at high temperatures in laboratory environments (Chomsky et al., 2004b). At low temperatures, meanwhile, *A. equina* is able to grow even when fed only once or twice a week (Chomsky et al., 2004a). As such, while 24 h of fasting should have eliminated the cost of digestion (Navarro et al., 1981), individuals probably still had flexibility in how they expended their energy at 13°C. This flexibility could have provided the scope for different personality types to employ different resource allocation strategies and for RMR to be positively correlated with growth (Killen, 2014). Shy individuals may thus have prioritised growth to a greater degree than bold individuals at low temperatures, both because of the greater flexibility in resource allocation that those temperatures afforded, and because low risk of predation from ectothermic predators at those temperatures (e.g. Twardochleb et al., 2020; Yamane and Gilman, 2009) should have allowed them to forage more freely. Alternatively, the move from holding tanks to respirometry chambers, or the slight disturbance in the chambers caused by the intermittent-flow cycle itself, might have caused disproportionate stress responses in shyer animals as compared with their bolder conspecifics, driving their higher RMRs at 13°C (e.g. Martins et al., 2011). In turn, shy individuals may already have been maximally stressed at that temperature and thus had little capacity to further increase their RMR at higher temperatures. Future studies could investigate the relationships between growth rate, dry mass and IRT to see whether shy individuals do grow faster than bold individuals at non-stressful temperatures, and what implications this has for selection.

In contrast to 13°C, individuals that were bolder at either temperature exhibited higher RMRs when under thermal stress than those that were shyer. One possible mechanistic underpinning this relationship could have involved shy individuals suppressing their RMRs to a greater degree than bold individuals at stressful high temperatures, in preparation for cooler ‘recovery periods’ in the future (Schulte et al., 2011). Temperature-driven adaptive metabolic suppression (either limiting the increase or even decreasing metabolic rates rapidly by curbing processes with high energy requirements; Schulte et al., 2011) appears to be specific to intertidal and shallow-water invertebrates, having been so far documented in gastropods (McMahon et al., 1995), molluscs (Vajedsamiei et al., 2021) and freshwater crustaceans (Glazier et al., 2020). Animals employing this strategy may be equipped during heatwaves to avoid their energetic demands exceeding their available energy supply but, as heatwaves become more frequent, suppression strategies could place individuals at a selective disadvantage by creating energetic deficits which they are unable to fulfil during progressively shorter recovery periods (Pörtner, 2012). Although they were of varying personality types, those individuals suppressing their RMRs most in our sample could have been the four that failed to increase their RMRs at all at 21°C (e.g. Vajedsamiei et al., 2021). It is also possible that these individuals had surpassed their metabolic thermal maximum (Schulte et al., 2011). Although the high RMRs of all four of these individuals at 13°C might suggest this is plausible, it would seem unlikely that any of them had surpassed this threshold given that lethal temperatures for other UK populations of *A. equina* are at least 2°C higher than the 21°C used in this experiment (Maskrey et al., 2020). Future studies could investigate molecular changes associated with metabolic suppression (Richier et al., 2008; Tomanek and Somero, 1999) to address these two possibilities directly.

The effects of individual and morphotypic variation on the plasticity of RMR could shed further light on its relationship with IRT. There was a general trend towards higher RMRs at 21°C, which remained unexpectedly consistent between the two morphotypes. Importantly, however, as predicted, this response was not ubiquitous, possibly indicating variation among individuals in strategies for dealing with extreme high temperatures. One potential explanation is that some individuals may compensate for increased RMR induced by higher temperatures by reducing activity. This could have led to the observed negative correlation between IRT and RMR at 21°C. After considering the available evidence, however, we believe that such a compensatory reduction in activity may be unlikely. Foraging tentacle expansion is the main energetically costly activity these anemones undertake (Griffiths, 1977) and IRT is thus not only an excellent proxy for boldness but also should act as a proxy for activity. As such, if a change in activity was driving the change in RMR, IRT and RMR at 21°C should have been closely correlated, a pattern that is not borne out by the data presented here. Indeed, RMR at 21°C was more closely correlated with IRT at 13°C than at 21°C, which does not suggest a close association between an individual's current level of activity and its RMR. Previous research also shows differences in how morphotypes change their IRTs, and thus their activity, across temperatures (Maskrey et al., 2020). These discrepancies were, unexpectedly, not reflected in morphotypic patterns of RMR in these data, further indicating that changing activity was not driving changing RMR. Instead, it seems more likely that relationships between IRT and RMR were at least partly driven by boldness-associated physiological differences, either via varied growth strategies at non-stressful temperatures and RMR suppression at high temperatures as we speculate, or by other mechanisms.

The patterns elucidated here may have important selective implications for *A. equina* as the frequency of heatwaves continues to increase (IPCC, 2013, 2018)*.* At the broader genotypic scale, the red morphotype, associated with the high shore, may be at a selective advantage as predicted. Not only do individuals of the red morphotype, in contrast to their green counterparts, lower their IRTs (Maskrey et al., 2020, 2021) at high temperatures, which should provide better mitigation against metabolic challenges under higher energetic demand (Griffiths, 1977; Navarro et al., 1981), but these data show that they also exhibit lower overall RMRs than green individuals, potentially further accentuating this increased efficiency. At the level of individuals, the directionality of any selective implications is less clear. It is possible that shy animals exhibit a more robust strategy than bold ones under current environmental conditions, up-regulating their metabolism when not under heat stress to invest in increased body mass, and conserving energy when they are. Bold individuals, meanwhile, may be at greater risk of perishing during heatwaves, by investing less in growth at lower temperatures and exposing themselves to greater metabolic requirements and increased risks of predation when under heat stress. Equally, shy animals could be at a selective disadvantage if they are unable to recoup any energetic deficit incurred during metabolically supressed periods, which could become increasingly likely as the frequency of heatwaves increases and recovery periods become more infrequent. Future work should investigate these possibilities directly, and incorporate life-history characteristics such as survival or fecundity. This could provide clearer information on not only the vulnerability of different *A. equina* individuals and genotypes to heatwaves but also that of other species, as the heterogeneous nature of the seashore leads many intertidal ectotherms to display intraspecific variation in behavioural and physiological responses to stressful high temperatures (Briffa et al., 2013; Chapperon et al., 2016; Dias et al., 2018; Maskrey et al., 2021).

Our measures of IRT resulted in censored data, given that there was a maximum cut-off time for shy individuals. Such data are common among behavioural studies (e.g. Stamps et al., 2012), but nevertheless they complicated our analysis of the relationships between RMR and IRT. We attempted a variety of approaches but the ability of current statistical packages to deal with these sorts of data were limited (although future developments may provide ways forward). Though our final statistical design was able to produce appropriate models, the likes of which are utilised in behavioural studies across the literature, there are concerns over its ability to deal with censored values (Stamps et al., 2012). As such, these results may need to be considered with care but we are confident that they are reflective of the biology of these organisms.

This study shows that associations between RMR and boldness in *A. equina* are highly temperature dependent, and that variation in trait plasticity could be part of the reason why evidence for POLS is so inconsistent. Individual differences were key drivers of how RMR changed between temperatures in this study, while morphotypic differences remained consistent, suggesting changing associations between RMR and boldness were being driven by individual-level plasticity. By measuring the boldness and metabolism of animals living in heterogeneous environments under different contexts, and adding a life-history component, future work could draw a clearer picture of POLS, gain insights into how different populations might respond to more regular climate change-induced heatwaves, and facilitate a more comprehensive understanding of the adaptive value of different personality types under climate change.

## Supplementary Material

10.1242/jexbio.244662_sup1 Supplementary information
