# Supplementary material for: Differential metabolic responses in bold and shy sea anemones during a simulated heatwave
Source: J Exp Biol. 2024 Feb 7;227(3):jeb244662. doi: 10.1242/jeb.244662 (PMC10912810; doi:10.1242/jeb.244662)
Supplement: Supplementary information [file jexbio-227-244662-s1.pdf]

Supplementary Materials and Methods

1.1 Metabolic apparatus and additional measurement detail

Automated intermittent-flow cycles were precisely controlled by a National Instruments C Series Counter Input Module (National Instruments, Austin, USA). Water in the respirometry tank was fully aerated throughout testing by a Tetra Second Nature Whisper 800 air pump (Tetra GMBH, Melle, Germany). Each chamber contained a central 6 x 6 x 6 cm polypropylene mesh cage containing a magnetic stir bar, which was controlled by an IKAMAG multi-position magnetic stirrer plate (IKA England LTD, Oxford, UK) positioned under the respirometry tank. For the ambient 13°C measurements, the temperature within the respirometry tank was maintained to a precision of ± 0.2°C by the laboratory's temperature control. At 21°C, the temperature of the tank was maintained to a precision of ± 0.3°C; a waterproof DS1820B temperature sensor (Maxim Integrated Products, Sunnyvale, USA) was connected to an Arduino Uno (Arduino LLC, Boston, USA) and used to control a LightwaveRF wireless control plug socket (LightwaveRF, Birmingham, UK) which was itself connected to an Eheim thermocontrol 300W heater (Eheim, Deizisau, Germany) submerged in the tank. During experimentation, the temperature in the respirometry tank was recorded at half hour intervals, taking measurements directly from the temperature sensor using the serial monitor within the Arduino software. Oxygen concentration, and by extension, oxygen consumption, was measured at second intervals using two PyroScience Firesting O_2 dissolved oxygen sensors (PyroScience GmbH, Aachen, Germany) each connected to three PyroScience 3mm diameter robust oxygen probes fed directly into chambers. Probes were calibrated at the start of each day of metabolic testing.

Table S1. Full checklist of essential information on our aquatic respirometry methods (from: Killen et al., 2021)

Number	Criterion and Category	Response	Value (where required)	Units
	**EQUIPMENT, MATERIALS, AND SETUP**			
1	Body mass of animals at time of respirometry	Pedal disc diameter (PDD) threshold used as no reliable, non-invasive measure of anemone volume	20	mm
2	Volume of empty respirometers	422.5ml		
3	How chamber mixing was achieved	Magnetic stirrer		
4	Ratio of net respirometer volume (plus any associated tubing in mixing circuit) to animal body mass	N/a, no reliable measure of live anemone volume so threshold PDD used		
5	Material of tubing used in any mixing circuit	PVC		
6	Volume of tubing in any mixing circuit	N/a		
7	Confirm volume of tubing in any mixing circuit was included in calculations of oxygen uptake	N/a		
8	Material of respirometer (e.g. glass, acrylic, etc.)	Glass, Polypropylene lid		
9	Type of oxygen probe and data recording	Pyroscience Firesting O_2		
10	Sampling frequency of water dissolved oxygen	1s		
11	Describe placement of oxygen probe (in mixing circuit or directly in chamber)	Directly		
12	Flow rate during flushing and recirculation, or confirm that chamber returned to normoxia during flushing	Chamber returned to normoxia (Appendix 1.3)		
13	Timing of flush/closed cycles			
14	Wait (delay) time excluded from closed measurement cycles	>60s		
15	Frequency and method of probe calibration (for both 0 and 100% calibrations)	Daily, calibrated to 100% dissolved oxygen saturation		
16	State whether software temperature compensation was used during recording of water oxygen concentration	Yes, standardised to temperature of treatment, not to real-time fluctuation		
	**MEASUREMENT CONDITIONS**			
17	Temperature during respirometry	Variable	13/21	ºC
18	How temperature was controlled	Arduino	± 0.3	ºC
19	Photoperiod during respirometry	12:12h		
20	If (and how) ambient water bath was cleaned and aerated during measurement of oxygen uptake (e.g. filtration, periodic or continuous water changes)	Periodic water changes and bleach sterilisation. Air stone for aeration.		

Journal of Experimental Biology • Supplementary information

21	Total volume of ambient water bath and any associated reservoirs	$61640cm^3$ (67cmx46cmx20cm)
22	Minimum water oxygen dissolved oxygen reached during closed phases	>80%
23	State whether chambers were visually shielded from external disturbance	Yes, opaque shield around water bath
24	How many animals were measured during a given respirometry trial (i.e. how many animals were in the same water bath)	Five
25	If multiple animals were measured simultaneously, state whether they were able to see each other during measurements	N/a
26	Duration of animal fasting before placement in respirometer	>24h
27	Duration of all trials combined (number of days to measure all animals in the study)	24 days
28	Acclimation time to the laboratory (or time since capture for field studies) before respirometry measurements	48h x 2
	**BACKGROUND RESPIRATION**	
29	Whether background microbial respiration was measured and accounted for, and if so, method used (e.g. parallel measures with empty respirometry chamber, measurements before and after for all chambers while empty, both)	Yes, parallel measures
30	If background respiration was measured at beginning and/or end, state how many slopes and for what duration	N/a
31	How changes in background respiration were modelled over time (e.g. linear, exponential, parallel measures)	Parallel measures
32	Level of background respiration (e.g. as a percentage of SMR)	Temperature and trial-dependent
33	Method and frequency of system cleaning (e.g. system bleached between each trial, UV lamp)	System and chambers bleached between each trial
	**STANDARD OR ROUTINE METABOLIC RATE**	
34	Acclimation time after transfer to chamber, or alternatively, time to reach beginning of metabolic rate measurements after introduction to chamber	12h
35	Duration over which metabolic rate was estimated	38 mins x 6
36	Value taken as SMR/RMR (e.g. quantile, mean of lowest 10 percent, mean of all values)	N/a, individual slopes recorded for analyses
37	Total number of slopes measured and used to derive metabolic rate (e.g. how much data were used to calculate quantiles)	N/a, individual slopes recorded for analyses

Journal of Experimental Biology • Supplementary information

38	Whether any time periods were removed from calculations of SMR/RMR (e.g. data during acclimation, periods of high activity [e.g. daytime])	No data were recorded during acclimation / attachment
39	r^2 threshold for slopes used for SMR/RMR (or mean)	0.9
40	Proportion of data removed due to being outliers below r-squared threshold	0%
	**MAXIMUM METABOLIC RATE**	
41	When MMR was measured in relation to SMR (i.e. before or after)	N/a
42	Method used (e.g. critical swimming speed respirometry, swim to exhaustion in swim tunnel, or chase to exhaustion)	N/a
43	Value taken as MMR (e.g. the highest rate of oxygen uptake value after transfer, average of highest values)	N/a
44	If MMR measured post-exhaustion, length of activity challenge or chase (e.g. 2 min, until exhaustion, etc.)	N/a
45	If MMR measured post-exhaustion, state whether further air-exposure was added after exercise	N/a
46	If MMR measured post-exhaustion, time until transfer to chamber after exhaustion or time to start of oxygen uptake recording	N/a
47	Duration of slopes used to calculate MMR (e.g. 1 min, 5 min, etc.)	N/a
48	Slope estimation method for MMR (e.g. rolling regression, sequential discrete time frames)	N/a
49	How absolute aerobic scope and/or factorial aerobic scope is calculated (i.e. using raw SMR and MMR, allometrically mass-adjusted SMR and MMR, or allometrically mass-adjusting aerobic scope itself)	N/a
	**DATA HANDLING AND STATISTICS**	
50	Sample size	60 overall, 20 control, 40 crossed-over
51	How oxygen uptake rates were calculated (software or script, equation, units, etc.)	respr R package followed by conversions to mgO_2/h
52	Confirm that volume (mass) of animal was subtracted from respirometer volume when calculating oxygen uptake rates	No, size was standardised to PDD as far as possible
53	State whether analyses accounted for variation in body mass and describe any allometric mass-corrections or adjustments	Yes, all analyses incorporated dry-weight as a fixed effect

Journal of Experimental Biology • Supplementary information

1.2 Example Slopes and conversion

Raw oxygen concentration was measured in % O_2 concentration and chamber volume less the volume of the magnetic stirrers, was 422.5ml. Because wet-weight and anemone volume could not be reliably measured, the volume of anemones was not corrected for. Initial slopes were thus measured in %O_2/s/422.5ml. These were first converted, using temperature-specific concentration conversion tables (Boyd & Pillai, 1985), to mgO_2/s/422.5ml. These values were then multiplied by 3600 to give the slopes in mgO_2/h/422.5ml, before being further multiplied by 0.4225 to give the values in mgO_2/ h.

In the majority of measured slopes of oxygen consumption a steady, linear decline in oxygen concentration was observed after an initial wait period of at least one minute (Svendsen et al., 2016; Figure S1), but there were some instances where slopes showed more noise. The respR package provides mitigation for this by calculating rolling regressions (Harianto et al., 2019), allowing accurate slope estimation even with a substantial degree of noise in the data around the gradient of the slope (Figure S2). In some instances, there were periods of noise which influenced the results of the rolling regression (Figure S3). In these instances, where possible, the longest, most stable, periods of linear decline of oxygen concentration were used in slope calculations (White et al., 2016). Where this was not possible, the slope was discounted from the dataset. One individual, from the H-L treatment and of the green morphotype, was discounted from all analyses as it showed no measurable slopes at 21°C. With this individual removed, of 826 oxygen consumption measurements (including blanks), 42 were measured using substantially truncated slopes. Of 708 anemone consumption measurements, nine were discounted from analyses.

Journal of Experimental Biology • Supplementary information

1.3 Statistical detail

Model Specification

Univariate models were fitted over uninformative Inverse-Wishart priors and run for 420000 iterations with a 20000-iteration initial burn-in period and a thinning interval of 100. Bivariate and multivariate models were fit similarly, but over 1000000 iterations with a 50000-iteration burn-in. To ensure successful convergence visual inspection of convergence and autocorrelation plots was carried out. To further confirm this, Heidelberger-Welch and Gelman-Rubin diagnostic tests were run on all models. Comparative models were also run over alternative, parameter-expanded priors, to ensure robustness to different prior specifications. Model estimates and deviance-information criteria (DIC; Spiegelhalter et al., 2002) did not differ meaningfully between the different specifications.

Model choice random slopes

Two models were initially run on our full dataset. A random intercepts model, not taking into account individual-level variation in metabolic plasticity to temperature, and a random slopes model. Once assumptions and convergence of both models had been checked, the two models' DIC values (Spiegelhalter et al., 2002) were compared. The DIC of the random slopes model (DIC = 666) was dramatically lower than that of the random intercepts model (DIC = 1110), indicating very clearly that the former, incorporating a random slope effect, was a better fit to the data than the latter.

The relationship between RMR and dry weight

To explore the relationship between size and RMR, a further bivariate model was run where RMR and dry weight were set as the response variables. Fixed and random effects were the same as the bivariate model above but with temperature set as an extra fixed effect. This confirmed a strong positive correlation between RMR and dry weight (r = 0.72, 95%CI = 0.57, 0.86; Figure S4). The size

of the correlation found here is in line with previous work in *A. equina* (Navarro et al., 1981) and also indicates that RMR in this species, when investigated in relation to dry weight, follows a scaling pattern roughly in line with the ¾ power relationship discussed in Brown et al (2004).

SI: References

Boyd, C. E., & Pillai, V. K. (1985). Water Quality Management in Aquaculture. *CMFRI Special Publication 22*, 1–44.

Brown, J. H., Gillooly, J. F., Allen, A. P., Savage, V. M., & West, G. B. (2004). Toward a metabolic theory of ecology. *Ecology*, *85*(7), 1771–1789. https://doi.org/10.1890/03-9000

Harianto, J., Carey, N., & Byrne, M. (2019). respR—An R package for the manipulation and analysis of respirometry data. *Methods in Ecology and Evolution*, *10*(6), 912–920. https://doi.org/10.1111/2041-210X.13162

Killen, S. S., Christensen, E. A. F., Cortese, D., Závorka, L., Norin, T., Cotgrove, L., Crespel, A., Munson, A., Nati, J. J. H., Papatheodoulou, M., & McKenzie, D. J. (2021). Guidelines for reporting methods to estimate metabolic rates by aquatic intermittent-flow respirometry. *Journal of Experimental Biology*, *224*(18), jeb242522. https://doi.org/10.1242/jeb.242522

Navarro, E., Ortega, M. M., & Madariaga, J. M. (1981). Effect of body size, temperature and shore level on aquatic and aerial respiration of Actinia equina (L.) (Anthozoa). *Journal of Experimental Marine Biology and Ecology*, *53*(2–3), 153–162. https://doi.org/10.1016/0022-0981(81)90016-2

Spiegelhalter, D. J., Best, N. G., Carlin, B. P., & Van Der Linde, A. (2002). Bayesian measures of model complexity and fit. *Journal of the Royal Statistical Society. Series B: Statistical Methodology*, *64*(4), 583–616. https://doi.org/10.1111/1467-9868.00353

Svendsen, M. B. S., Bushnell, P. G., & Steffensen, J. F. (2016). Design and setup of intermittent-flow respirometry system for aquatic organisms. *Journal of Fish Biology*, *88*(1), 26–50. https://doi.org/10.1111/jfb.12797

White, S. J., Kells, T. J., & Wilson, A. J. (2016). Metabolism, personality and pace of life in the Trinidadian guppy, Poecilia reticulata. *Behaviour*, *153*(13–14), 1517–1543. https://doi.org/10.1163/1568539x-00003375

Journal of Experimental Biology • Supplementary information

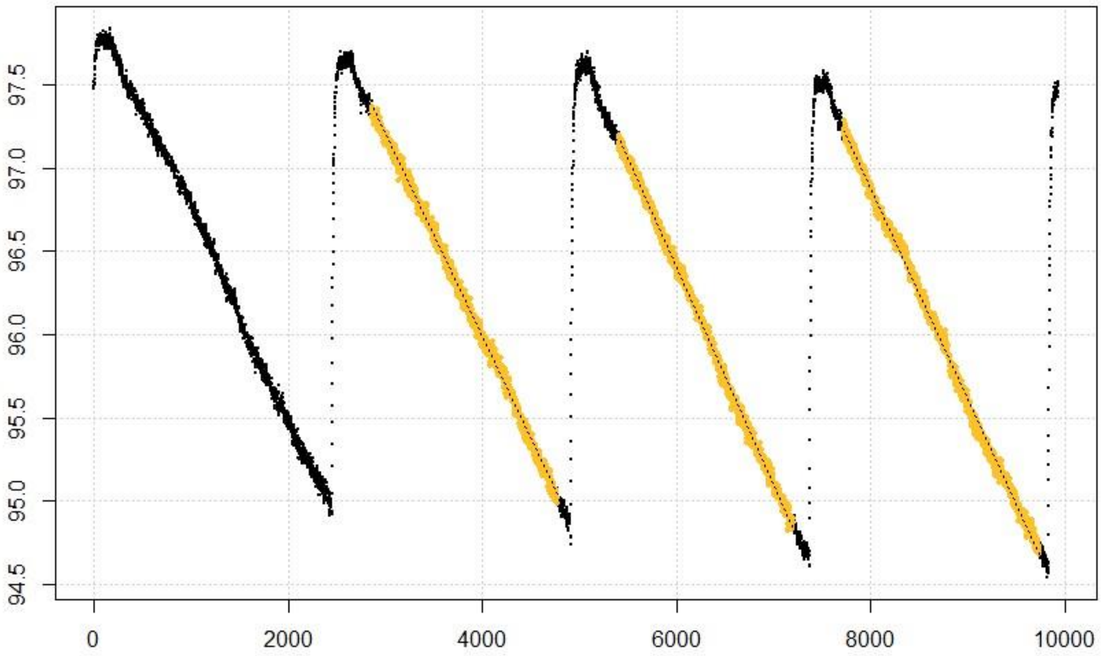

Fig. S1. Example of metabolic slopes with no fluctuation or truncation.

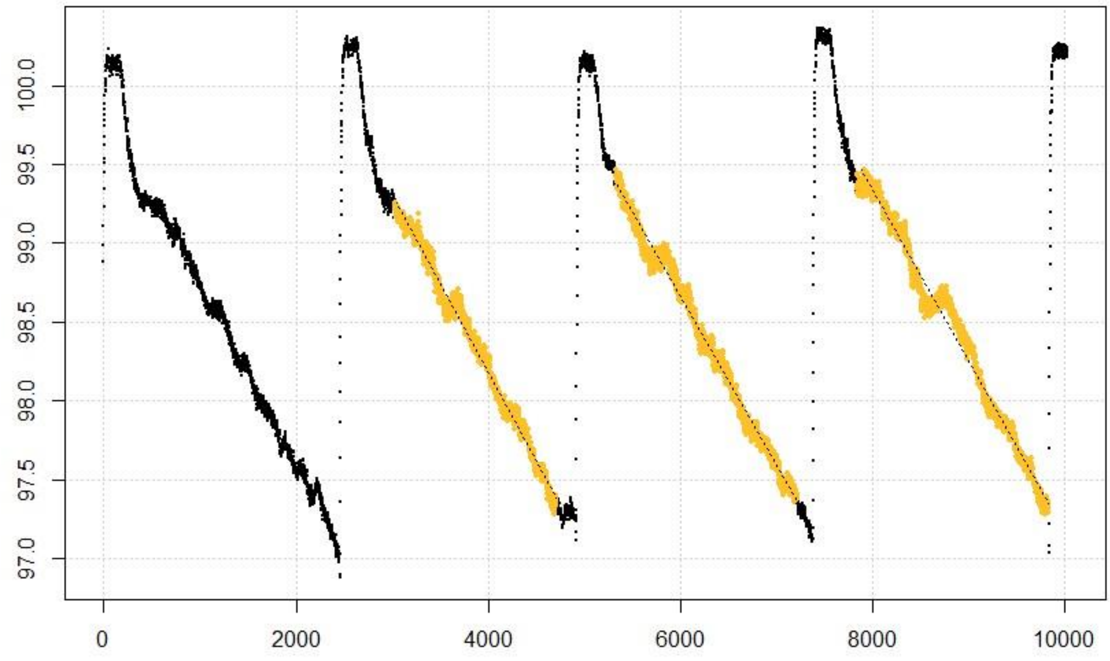

Fig. S2. Example of metabolic slopes where fluctuations could be dealt with by rolling regression.

Journal of Experimental Biology • Supplementary information

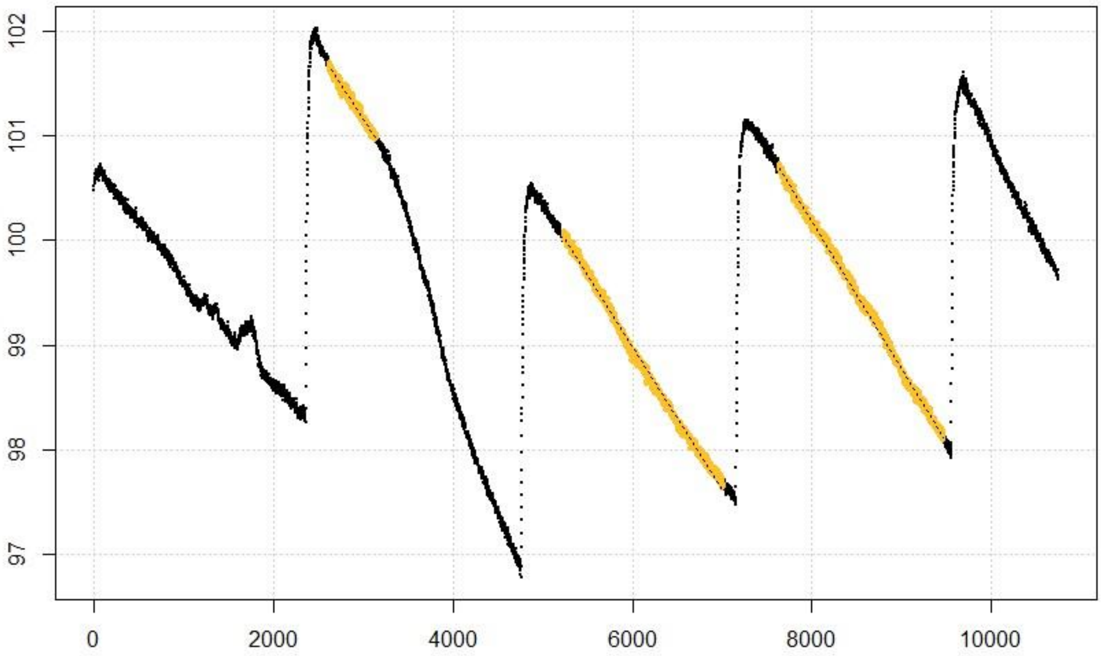

Fig. S3. Example of a metabolic slope where a large degree of truncation was required.

Journal of Experimental Biology • Supplementary information

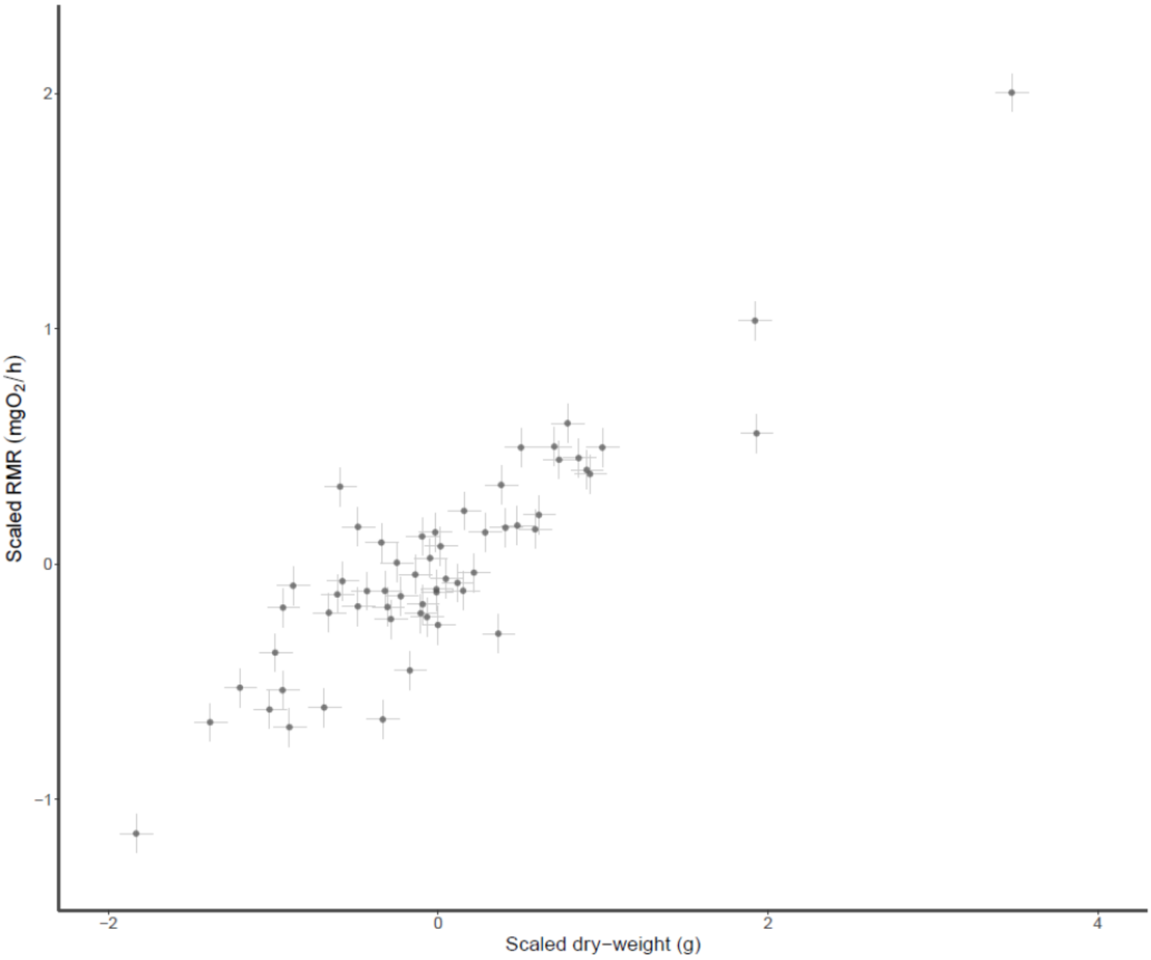

Fig. S4. The relationship between the posterior mode estimates (Bayesian BLUPS) of individual RMR and individual dry weight, derived from a bivariate mixed effects model run on the full dataset (n=60).
